# Micro and Nano Drug Delivery Systems for the Treatment of Oral Mucositis: A Review

**DOI:** 10.3390/pharmaceutics17081025

**Published:** 2025-08-07

**Authors:** Luciana Ângela Soares Maia, Tâmara Thaiane Almeida Siqueira, Carlos Alberto Arcelly Santos Bezerra, Jéssica Horana Pereira de Farias, Elquio Eleamen Oliveira

**Affiliations:** Laboratory of Synthesis and Drug Delivery, Paraiba State University, Rua Horácio Trajano de Oliveira, s/n, Cristo Redentor, João Pessoa 58075-540, PB, Brazil; lucianamaia74@gmail.com (L.Â.S.M.); thaianesiqueira@gmail.com (T.T.A.S.); c.alberto7@gmail.com (C.A.A.S.B.); jessica.horana@aluno.uepb.edu.br (J.H.P.d.F.)

**Keywords:** dosage form, drug delivery, oral mucositis, microsystems, nanosystems, treatment

## Abstract

Oral mucositis (OM) is a severe inflammatory condition of the oral mucosa that is commonly associated with cancer therapies. Traditional treatments typically have limited efficacy and significant side effects, necessitating alternative approaches. Nanobased drug delivery systems (DDSs) present promising solutions, enhancing therapeutic outcomes while minimizing side effects. This review aims to evaluate the use of nanobased DDSs to treat OM. To reach these aims, an extensive literature review was conducted using the following databases: BVS, PubMed, Scopus, and Web of Science. The search strategy included the keywords “microparticles,” “nanoparticles,” “drug delivery system,” “oral mucositis,” “therapy,” and “treatment,” combined with the Boolean operators “AND” and “OR.” After applying filters for language, relevance, full-text availability, exclusion of review articles, and removal of duplicates, a total of 32 articles were selected for analysis. Of the 32 studies included in this review, 25 employed polymeric micro- or nanosystems for the treatment of OM. Regarding the stage of investigation, 10 studies were conducted in vitro, 16 were conducted in vivo, and 6 corresponded to clinical trials. Compared with conventional drug delivery approaches, most of these studies reported improved therapeutic outcomes. These findings highlight the potential of nanosystems as innovative strategies for enhancing OM treatment. Nonetheless, challenges in large-scale manufacturing, including reproducibility and safety, and the limited number of clinical trials warrant careful consideration. Future research with larger clinical trials is essential to validate these findings and effectively guide clinical practice.

## 1. Introduction

**Figure 1:**
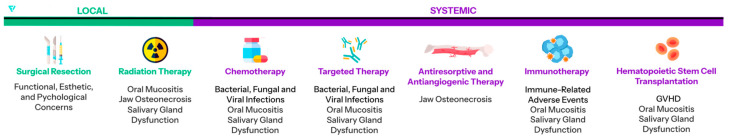
Methods of cancer therapy and oral health considerations.

Oncologic therapies, while so important in the fight against cancer, can have significant side effects on oral health. These treatments, including immunotherapy, radiotherapy, and chemotherapy, often lead to oral complications such as oral inflammation and ulcers. In particular, head and neck radiotherapy and chemotherapy are noted for having a significant negative impact on the patient’s oral health [1]. The most common complication is oral mucositis (OM), which is characterized by severe inflammation and the formation of painful ulcers [2]. In addition, these treatments can temporarily impair salivary gland function, causing xerostomia, which increases the risk of infection, cysts, and other oral complications [3]. The major therapeutic approaches used to treat cancer are shown in Figure 1, along with the oral complications associated with each approach.

### 1.1. Oral Mucositis

OM, induced by radiotherapy and chemotherapy, is characterized by the appearance of erythema, ulcers, and pseudomembranes and compromises functions such as chewing, swallowing, and articulation [4,5]. This condition can expose patients to opportunistic infections and requires symptomatic and preventive interventions. Treatment options include analgesics, local anesthetics, antifungals, and antivirals, as well as care such as applying gel before and after sessions, avoiding irritating foods, and rinsing with saline solution, which all help reduce the severity of symptoms. Avoiding mouthwashes containing alcohol, coffee, or alcoholic beverages is recommended, as they can aggravate inflammation [6].

Low-potency laser therapy has an anti-inflammatory effect and stimulates healing [7]. Cryotherapy, through ice fragments in the oral cavity, helps decrease the inflammatory process [8]. Other methods include applying L-glutamine or vitamin E topically to lesions and using chamomile tea to relieve mucosal discomfort [9].

Researchers have dedicated themselves to improving drug release techniques on the oral mucosa and developing strategies based on organic micro- and nanosystems. These systems provide control over the release velocity of therapeutic agents, ensuring greater precision in local dosing and reducing systemic adverse effects [10]. These systems include emulsions, suspensions, micelles, liposomes, hydrogels, nanofibers, dendrimers, and polymeric nanoparticles (NPs), which aim to protect the drug and optimize its bioavailability. Recent papers have indicated that this approach can contribute to the treatment of OM by increasing the drug concentration in the affected region, prolonging its effect, and accelerating the healing of injured mucosa, as observed with rebamipide nanocrystals and nanofibrils containing glutamine [11,12].

### 1.2. Micro- and Nanosystems

**Figure 2:**
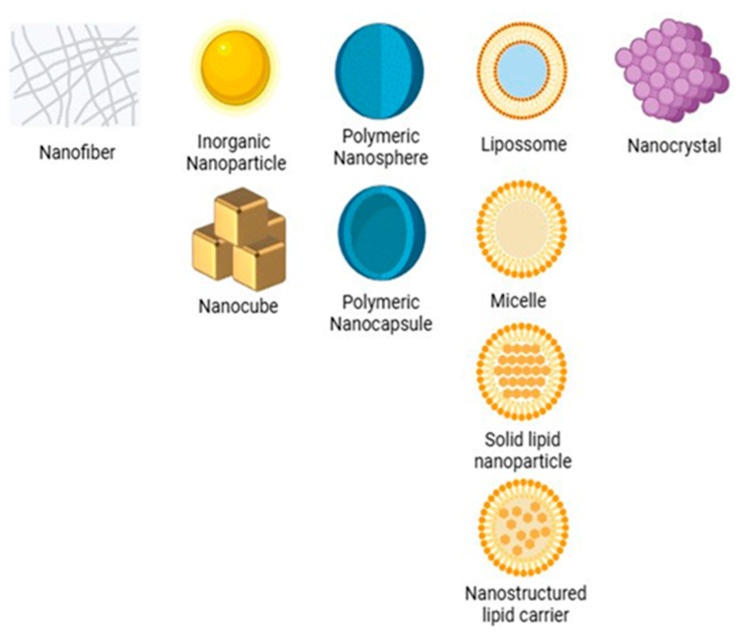
Classification and characteristics of nanoparticles used in drug delivery systems.

Micro- and nanosystems are technologies that operate on different scales. Microsystems use micrometric structures to encapsulate and release drugs in a controlled manner, while nanosystems can act at the molecular and cellular levels. This allows them to penetrate biological barriers and have a greater surface area for interacting with target cells [13]. Among nanosystems, NPs (1–1000 nm) stand out as their high surface-to-volume ratio facilitates drug delivery and interaction with epithelial cells [14]. They can take the form of nanocapsules, which confine an active ingredient in the nucleus and/or wall, or nanospheres, which disperse a drug in a polymeric matrix [15,16]. Other platforms include nanoemulsions stabilized by surfactants [17], micelles [18,19], phospholipid bilayer liposomes [20], and high-porosity nanofibers [21], as illustrated in Figure 2.

On the micrometer scale, microparticles are robust systems for controlled release. They range in size from 1 µm to 1000 µm and are classified as microspheres (hollow) and microcapsules (core and shell) [22]. When produced with biodegradable polymers such as chitosan and PLGA and sized between 1 and 10 µm, they ensure high encapsulation efficiency, deposition in specific areas, and sustained release [23].

### 1.3. Existing Reviews

Previous reviews have covered various treatments for oral mucositis. Colella et al. [24] analyzed randomized clinical trials and evaluated interventions such as anti-inflammatory drugs, low-level laser therapy, cryotherapy, herbal medicines, nutritional supplements and mucus protective agents, all of which showed potential to reduce lesion severity or incidence. However, results were heterogeneous and limited by small sample sizes and lack of standardization, indicating the need for further studies. Gholizadeh and collaborators [25] reviewed recent therapeutic approaches and noted that, although traditional methods such as hydration, nutritional support, and infection control remain in use, no definitive treatment protocol has been established. This review included emerging strategies such as low-intensity laser, epidermal growth factors, stem cells, hyaluronic acid, and metalloproteinase inhibitors, discussing their mechanisms of action and evidence of improvement in lesion severity, pain, and duration.

Other reviews, as presented by Ferreira et al. [26], address the use of natural products in the prevention and treatment of oral mucositis. This work examines the pathophysiological mechanisms and risk factors underlying oral lesions, as well as management strategies and the limitations of conventional therapies. The study highlights the therapeutic potential of natural compounds such as honey, Aloe vera, propolis, green tea, and curcumin, drawing on their anti-inflammatory, antioxidant, and wound-healing properties supported by preclinical and clinical evidence. The article also outlines future perspectives, including the development of drug delivery systems for bioactive natural products applied to oral mucosa.

Finally, some reviews focus on newer approaches to oral mucositis management. Choudhury et al. [27] examined gold NPs as therapeutic agents and covered mucositis pathophysiology, the role of inflammatory mediators such as NF-κB, and oxidative stress from reactive oxygen species. They described the anti-inflammatory, antioxidant, and antimicrobial properties of these particles, the challenges of toxicity, stability, and functionalization, and strategies to enhance clinical application. Review [28] evaluated nine studies, including six in vivo and three clinical trials, that investigated gold NPs, curcumin, silymarin, PLGA, and Eudragit nanofibers. These therapies demonstrated sustained release, improved oral adhesion, dose reduction, and fewer adverse effects compared to conventional treatments, highlighting the need for larger clinical trials to confirm their therapeutic efficacy.

### 1.4. Motivation and Objectives

This work is motivated to review and synthesize scientific evidence and advances in the use of micro- and NPs in oral mucosal therapy. This condition, often associated with anticancer treatments, causes severe inflammation and ulceration that impair vital functions, including swallowing, speaking, and chewing. Furthermore, mucositis increases susceptibility to opportunistic infections, posing an additional risk to patients and compromising their quality of life. Despite conventional treatment options, these strategies often have limitations and are unable to provide adequate symptom relief, highlighting the need for more innovative and effective therapeutic approaches.

In this context, a critical evaluation of the potential of diverse micro- and nanosystems is imperative to identify more precise and effective solutions for managing OM. The primary objective of this study was to conduct an integrative review of the utilization of micro- and nanosystems, including NPs, liposomes, and micelles, in the management of this condition. The objective is to analyze the available alternatives, the current state of research, and the pharmaceutical formulations based on organic nanosystems used in in vitro, in vivo, and clinical trials. This approach is unprecedented in the study of organic nanosystems for OM.

## 2. Materials and Methods

The PICo (problem, intervention, and context) search strategy was employed as a methodological framework to delineate the research question in the evidence-based bibliographic search. This methodology was selected to ensure that this research remained focused on the central elements of the topic of revision, thereby ensuring the accurate identification of relevant studies. Consequently, key terms related to the problem (oral mucositis), the intervention (drug delivery system involving nanoparticles and microparticles), and the specific treatment context were incorporated. Moreover, the PDF AI tool was used to conduct keyword-centric searches within the text of the articles. Despite this assistance, all articles were manually reviewed to verify their relevance and accuracy.

The initial search yielded 50 articles in BVS, 31 articles in PubMed, 117 articles in Scopus, and 34 articles in Web of Science. A filter based on language and document type was subsequently employed to eliminate articles that did not meet the criteria for complete and relevant articles. This process resulted in the removal of six articles from BVS, three from PubMed, forty-five from Scopus, and six from the Web of Science. The remaining 172 articles were subsequently reviewed in full. A filter based on language and document type was subsequently employed to remove nonrelevant records, resulting in 172 articles for full-text screening. At this stage, the following inclusion criteria were applied:

1. Use of a micro- or nanosystem (nonmetallic) as a drug delivery vehicle.
2. Articles published in English.
3. The full texts are available.

The following exclusion criteria were then applied:

1. Review articles, editorials, or conference abstracts.
2. Studies without clear methodological details or outcome data.
3. Duplicate records identified across databases.

After applying these criteria, 32 articles remained for inclusion in this review.

Furthermore, a risk of bias analysis was performed on all studies, which were grouped according to their research stage (in vitro, in vivo, and clinical trials) to apply specific criteria to each category. For in vitro studies, we verified whether the articles described the cellular model used to evaluate inflammatory markers, ensuring clarity regarding the methodology. For in vivo studies, we used the SYRCLE tool [29], a checklist based on domains such as randomization, blinding, and animal handling, developed to standardize the assessment of bias in preclinical animal research. In clinical trials, the Quality Assessment of Controlled Intervention Studies table, made available by the NIH was applied, which examines domains such as generation and concealment of the randomization sequence, blinding, losses to follow-up, and selective reporting of results.

## 3. Results

This section summarizes data from the selected articles, focusing on the application of micro- and nanosystems in drug delivery for the treatment of OM. The analysis includes the different types of micro- and nano DDS investigated, as well as the polymers used in their formulation, which are classified as natural or synthetic. Additionally, the studies were organized into separate tables according to the research stage: in vitro, in vivo, and clinical trials.

### 3.1. Main Polymers Used

When analyzing the distribution of the types of polymers used in the studies, nine studies used only synthetic polymers, whereas five studies used only natural polymers. However, most studies used a combination of natural and synthetic polymers, with a total of 11 publications. In addition, some studies did not use polymers in their formulations; these included [19,30,31,32,33,34]. These data suggest a slight preference for the use of synthetic materials in the treatment of OM.

**Figure 3:**
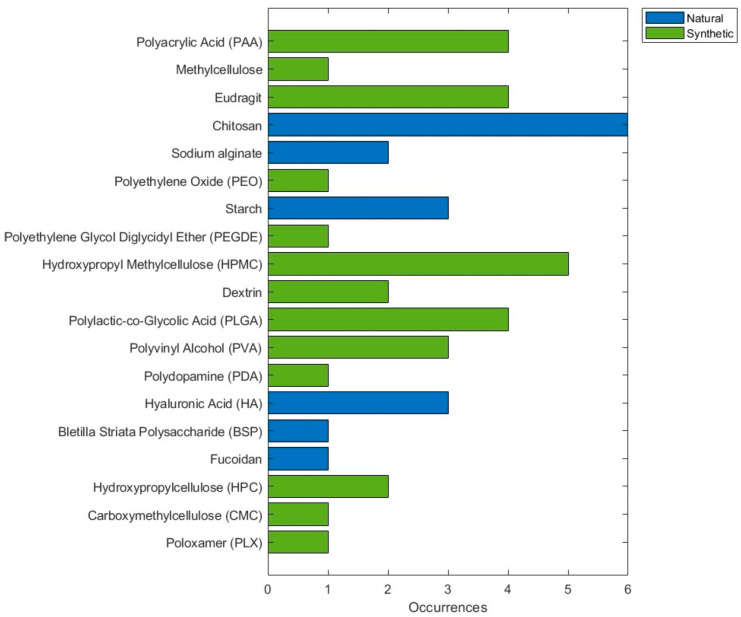
Frequency distribution of natural and synthetic polymers in drug delivery studies for oral mucositis. Natural polymers (blue) and synthetic polymers (green) are represented according to their occurrence.

Figure 3 illustrates the classification of polymers used in the studies, distinguishing between natural and synthetic polymers. Chitosan was the most common polymer, with a total of six occurrences, followed by another natural polymer, hyaluronic acid, with three occurrences. Among the synthetic polymers, hydroxypropylmethyl cellulose (HPMC) was the most used, with five occurrences, followed by polyacrylic acid (PAA), Eudragit, and polylactic-co-glycolic acid (PLGA), with four occurrences each.

### 3.2. In Vitro Studies

**Figure 4:**
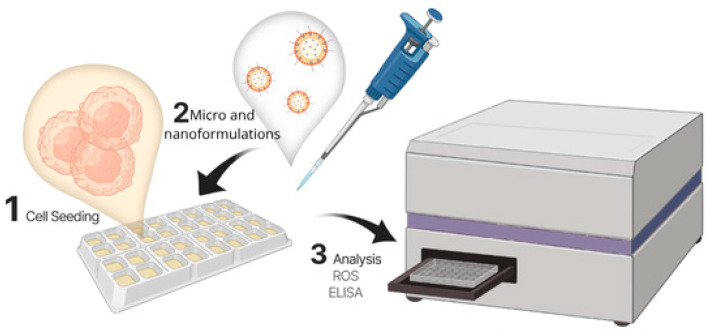
Flowchart of the steps of the in vitro assay for evaluation of micro- and nanosystems in oral mucositis: seeding of oral cells in a plate, treatment with micro- and nanoformulations, oxidative stress (ROS) analysis, and cytokine release (ELISA) analysis.

This section of this review compiles in vitro studies. Figure 4 illustrates the main stages of this type of trial. First, micro- or nanotherapy with the active drug is developed. Next, the formulation is characterized in terms of physical–chemical properties and morphology. Finally, the system is tested on oral keratinocytes or fibroblasts to verify the reduction in inflammatory markers and other cellular effects relevant to the treatment of OM.

Table 1 presents some of the relevant characteristics of the in vitro studies included in this review. The structure of the table is organized as follows: the first column presents the type of micro- or nanosystem developed; the second column specifies the polymer used as the basis for the formulation; the third column describes the therapeutic agent incorporated; the fourth column provides information on the cellular model used in the in vitro assays; the fifth column compiles the main results obtained; and the sixth column refers to the references of the analyzed studies. In total, ten articles, which address different experimental approaches for evaluating delivery systems applied to the treatment of OM, were included.

**Table 1:** Characteristics of in vitro studies with micro- and nanosystems for the treatment of OM.

Micro and Nano	Polymer	Therapeutic Agent	Cell Model	Main Results	Ref.
NPs	-	*P. indica* leaf extract	HO-1-N-1 (human oral squamous cell carcinoma)	*P. indica* NPs promoted the migration of oral mucosal cells, indicating potential in the treatment of oral wounds. The spray formulation was found to be stable and efficient for long-term use.	[31]
Microparticles	Chitosan	BZH	-	Chitosan microparticles with different ratios of chitosan to BZH showed sustained release and strong mucoadhesive adhesion, which is promising for long-term treatment of oromucosal conditions.	[35]
Nanofibers	Sodium alginate and PEO	Glutamine	-	Nanofibers showed promise as a mucoadhesive for OM, remaining stable at 4–25 °C, but less stable at higher temperatures. Glutamine release was gradual, with more than 85% released after 4 h.	[36]
Nanogels	Starch and PEGDE ^1^	Vancomycin (VNG)	-	VNG-loaded nanogels showed strong antibacterial activity against *Staphylococcus aureus*, *Streptococcus pyogenes*, and *Streptococcus mutans*, common pathogens in OM, showing promise for the treatment of oral infections.	[37]
Nanofibers	HA ^2^	Glycyrrhizin	RAW 264.7 macrophages and human epithelial cells	The glycyrrhizin-loaded nanofiber reduced proinflammatory cytokines and maintained 83% viability in 5-FU-treated cells, showing potential with artificial saliva.	[38]
Solid Lipid NPs (SLN)	HA and Carbopol^®^	N-cetylglucosamine (NAG)	-	The NAG-enriched SLN gel demonstrated mucoadhesion efficacy and preserved epithelial integrity, suggesting potential for healing and protecting mucositis-affected oral mucosa.	[39]
NPs	HPC-L, SL and SSL ^3^	Rebamipide	-	Rebamipide NPs were shown to be stable and adherent to the oral mucosa, providing localized, sustained release and potentially improving the quality of life of patients with stomatitis.	[40]
Microparticles	Eudragit	Chestnut shell extract rich in polyphenols	TR146 (squamous carcinoma of the oral mucosa)	Chestnut extract microparticles exhibited high antioxidant potential, biocompatibility, and effective polyphenol release, appearing promising for mucositis therapies.	[41]
Microparticles	Eudragit RS 30D	Actinidia arguta leaf extract	TR146 (buccal keratinocytes) and HSC-3 (tongue carcinoma)	Actinidia arguta microparticles had high phenolic content, strong antioxidant activity, and were well tolerated by oral epithelial cells, suggesting therapeutic potential for OM.	[42]
Hydrogels	HPMC, Carbopol^®^ and Sodium hyaluronate	Budesonide and Lidocaine	*-*	The formulation demonstrated significant mucoadhesive capacity and prolonged drug release suitable for the management of mucositis. Tablets showed better adhesion, while films were more resistant and customizable for application.	[43]

^1^ PEGDE: Polyethylene Glycol Diglycidyl Ether. ^2^ HA: Hyaluronic Acid. ^3^ HPC-L. SL and SSL: Hidroxi-propil cellulose with molecular weights of approximately 140,000, approximately 100,000, and approximately 40,000, respectively.

Notably, not all studies have detailed the cellular model used. In the case of reference [36], there is no specification of the cell type, but the use of freshly extracted sheep buccal mucosa, which was frozen and prepared for mucoadhesion assays, is mentioned. Saracoglu and colleagues [37] used a simulated salivary fluid containing α-amylase with the aim of reproducing the oral cavity environment. Cara et al. [39] adopted reconstituted human oral epithelium (RHO), which is composed of immortalized human epithelial cells cultured on a polycarbonate filter at an air–liquid interface, using a defined chemical medium.

Kawano and team [40] described the use of mucin derived from pig stomachs as a model of artificial mucosa, whereas Jiang and colleagues [35] applied an ex vivo model based on fresh chicken crop epithelium, which was chosen for yielding results consistent with in vivo studies on human buccal mucosa. Likewise, Campos and coworkers [43] did not report the cell model employed, and the in vitro work was confined to hydrogel characterization, mechanical testing, mucoadhesion assays, drug release profiling, and evaluation of rheological properties.

With respect to the base polymer, Buranasukhon and coauthors [31] exclusively indicated the use of Pluronic F-127, which was employed as a stabilizer in the NPs analyzed, without detailing the base polymer used.

### 3.3. In Vivo Studies

**Figure 5:**
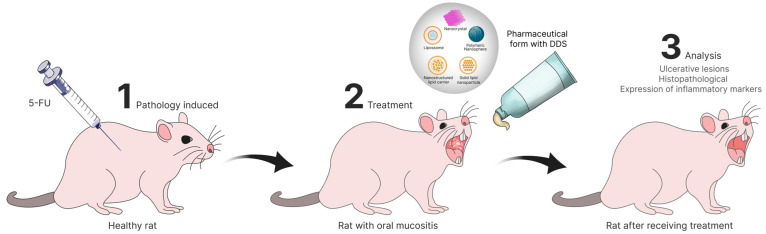
In vivo experimental model of oral mucositis in rats and treatment with a drug delivery system (DDS).

The reviewed in vivo studies employed a variety of animal models, including rats, hamsters, mice, albino rabbits, and beagle dogs, each offering specific advantages for assessing new formulations. OM was induced by methods such as acetic acid injection, administration of chemotherapeutic agents (for example 5-fluorouracil) or mechanical trauma, reproducing different degrees of lesion severity. Figure 5 shows a representative protocol in which a rat receives a single injection of 5-FU to trigger mucositis, followed by topical application of the micro- or nanosystem and subsequent evaluation of mucosal healing.

Table 2 presents the results of the selected articles for the in vivo studies. The structure follows the same format as in Table 1, with the only change being the replacement of the cellular model with the animal model. Thus, the columns remain organized as follows: type of micro- or nanosystem, polymer used as the base, incorporated therapeutic agent, animal model employed in the experiments, main results reported in the studies, and article references.

**Table 2:** In vivo studies using micro- and nanosystems for the treatment of OM.

Micro and Nano	Polymer	Therapeutic Agent	Animal Model	Main Results	Ref.
NPs	PLGA and Chitosan	Rebamipide	Mouse mucositis model	Chitosan-coated NPs significantly reduced ulcer area and treatment time compared with the control and uncoated-NPs groups. The coating contributed to greater efficacy in the treatment of OM.	[44]
Nanomicelles	Fucoid (FD)	Cannabidiol (CBD)	Mouse mucositis model	CBD-FD micelles significantly reduced ulcer area and inflammation vs. free CBD, showing excellent retention and healing in chemotherapy-induced lesions.	[45]
Nanocrystals	Carbopol^®^ and methylcellulose	Rebamipide	Hamster model	The rebamipide nanocrystal formulation improved healing and epithelial regeneration and reduced inflammation, likely through clathrin-mediated endocytosis.	[46]
Nanofibers	Eudragit and Chitosan	Human growth hormone (hGH)	Beagle dog model	hGH nanofibers with 0.5% chitosan fully regenerated ulcers, while the 1% chitosan version was less effective. The system allowed for controlled release of hGH, which enhanced healing.	[47]
Nanocubes	Starch and Dextrin	Apremilast	Kunming mice model	The formulation demonstrated potent anti-inflammatory and antioxidant capabilities, promoted healing of OM induced, demonstrated excellent adhesion and comfort, accelerated healing and promoted epithelial proliferation.	[48]
NPs	PLGA and PVA	Dexamethasone	Syrian golden hamsters	NPs reduced the severity of mucositis, with less inflammation and tissue damage, especially at a dose of 0.1 mg/kg. The use of PLGA allowed therapeutic efficacy with a reduced dose of dexamethasone, minimizing potential side effects.	[49]
NPs	PLGA and HPMC	Benzydamine (BZN)	Rabbit mucositis model	BZN-PLGA-NPs-HG reduced ulcer area in a mucositis model compared to the BZN-only and control groups. The PLGA-NPs-HG system had shorter healing time than other formulations.	[50]
NPs	PDA and PLGA	Dexamethasone	Rat model	The mussel-inspired mucoadhesive system enhanced film retention in a moist environment, tripling bioavailability and accelerating wound closure, showing potential for OM treatment.	[51]
NPs	HA and BSP	Triamcinolone acetonide (TA)	Rat mucositis model	Application of TA@MPDA ^1^-HA/BSP significantly reduced inflammation and accelerated healing of oral ulcers in rats, proving a promising alternative for treating oral inflammatory diseases	[52]
NPs	Carbopol^®^ and HPMC	Troxipide	Hamster mucositis model	The NP gel healed significantly faster than the microparticle gel, enhancing local drug absorption and retention. The therapeutic effect was mediated by the CME ^2^ endocytosis pathway, which proved effective in the mucositis model.	[53]
Nanoemulsion	-	Quercetin	Mouse mucositis model	Quercetin nanoemulsion reduced inflammation and tissue damage compared to the control group, with a remarkable protective effect against OM, showing potential for mucositis prevention.	[54]
Mucoadhesive microparticles	PVA ^3^	Indomethacin (IM)	Mouse oral mucosa retention model	The IM-NK(50) ^4^ gel formulation, using a specific type of PVA, showed the highest concentration of IM in the oral mucosa without a significant increase in plasma concentrations, indicating its potential to relieve pain without systemic effects.	[55]
Freeze-dried wafer	Chitosan, CMC ^5^, HPMC and PLX	Benzydamine hydrochloride (BZH)	Rat mucositis model	BZH-loaded wafer significantly reduced the severity of mucositis compared to controls, promoted accelerated healing, and decreased inflammatory cell infiltration.	[56]
Submicronized crystals of rebamipide	HPMC and HPC	Rebamipide	Rat model	Submicronized rebamipide crystals significantly reduced ulcers versus control. Intraoral administration led to higher mucosal drug concentration than intragastric delivery, enhancing therapeutic efficacy.	[57]
Nanofibers	Eudragit L100 and S100	Ketoprofen	Rabbit model	The formulation with ketoprofen (EL-NF) ^6^ relieved mucositis and promoted re-epithelialization in rabbits, remained adherent for up to 2 h, and provided sustained release and rapid healing.	[58]
Nanoemulsion	HPMC	Diclofenac and Lidocaine	Albino Rat and Mice Model	The formulation showed a strong anti-inflammatory effect (up to 87.99% reduction in edema) and effective analgesia in vivo, presenting a high acceptance rate and rapid pain relief.	[59]

^1^ TA@MPDA: Mesoporous polydopamine nanoparticles loaded with TAA. ^2^ CME: Clathrin-mediated endocytosis. ^3^ PVA: Polyvinyl alcohol. ^4^ IM-NK(50): Mucoadhesive microparticles containing indomethacin and 50 mg of polyvinyl acid-type NK-05R. ^5^ CMC: Carboxymethylcellulose. ^6^ EL-NF: Eudragit L100 nanofibers.

### 3.4. Clinical Studies

**Figure 6:**
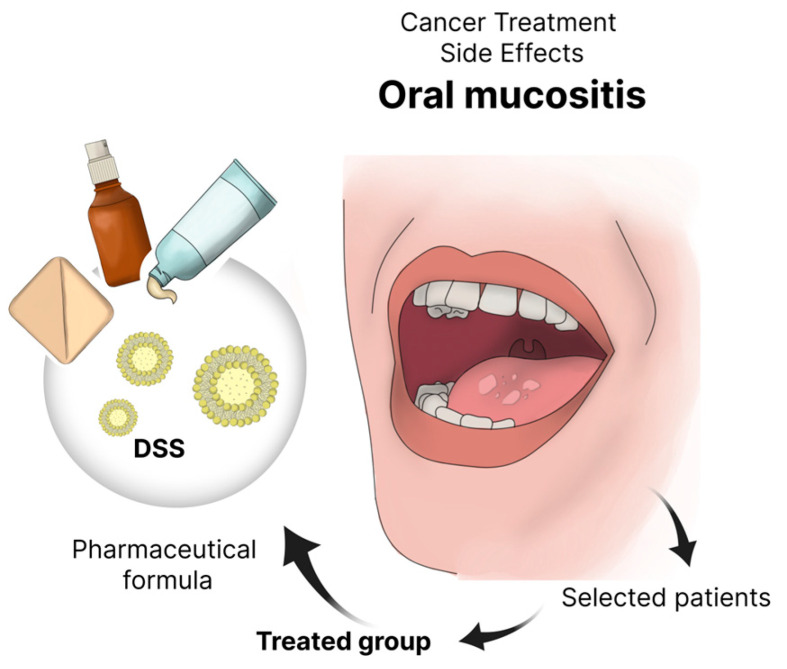
Clinical procedure to evaluate therapeutic results of micro- and nanobased drug delivery systems (DDS) for patients with head and neck cancer induction of oral mucositis by chemotherapy or radiotherapy.

Preclinical and clinical trials have shown that nanoformulations deliver significant benefits in treating OM, often outperforming conventional drugs in efficacy. Controlled release, biocompatibility, and patient acceptability emerge as key factors for success. To date, no microsystem-based therapy has reached the clinical stage. Figure 6 illustrates a typical clinical workflow for head and neck cancer patients: after chemotherapy or radiotherapy induces OM, the micro- or nanoformulated DDS is applied to the lesions, and treatment outcomes are then evaluated.

Table 3 presents the clinical studies identified in this review. The columns corresponding to the polymer and the model were replaced by concentration, dose, and frequency of administration, which are considered more relevant in the clinical context. Curcumin was the most frequently used therapeutic agent, appearing in four of the seven studies analyzed [30,32,33,34], whereas micelles were the most recurrent DDS, present in four studies [19,32,33,34].

**Table 3:** Clinical studies with micro- and nanosystems for the treatment of patients with OM.

Micro and Nano	Therapeutic Agent	Concentration	Dose and Frequency	Main Results	Ref.
Micelles	Silymarin	70 mg/5 mL	5 mL, 3× daily for 6 weeks	The nanosilymarin formulation slightly reduced mucositis progression in four weeks but was not statistically significant. It was well tolerated, although some reported unpleasant taste and mild gastrointestinal reactions.	[19]
NPs	Curcumin	NPs with 0.1% curcumin	10 mL, 3× daily (without dilution), for 7 days	The use of curcumin mouthwash reduced the risk of mucositis onset by 50% and delayed its onset by an average of two weeks compared to the control group (benzydamine), with less severity observed.	[30]
Micelles	Curcumin	80 mg of curcumin in micelles per capsule	1 capsule a day during radiotherapy	Curcumin micelles reduced mucositis severity during radiotherapy, halving grade 4 incidence vs. control, proving effective for prevention and management.	[32]
Micelles	Curcumin	0.1% *w*/*v* for mouthwash; 40 mg curcuminoids for curcumin capsule	10 mL, 3× daily for 21 days for mouthwash; 1 capsule a day for 21 days	Curcumin, orally or topically, significantly reduced pain and inflammation in radiation-induced mucositis. Over 33% using mouthwash and 15% using capsules remained ulcer-free. No significant differences occurred between forms.	[33]
Micelles	Curcumin	80 mg of curcumin in micelles per capsule	Two capsules a day, after meals, for 7 weeks	Patients receiving curcumin micelles had less mucositis progression and pain over seven weeks. Its anti-inflammatory and antioxidant effects helped control mucositis, especially in chemotherapy-only patients.	[34]
Nanogel	Doxepin	0.2% *w*/*v* chitosan with 5 mg/mL doxepin	Nanogel application on the lesions with a cotton swab, 4× a day	Chitosan nanogel with doxepin reduced markers of inflammation and pain more effectively than conventional treatments, demonstrating positive clinical results for the treatment of mucositis.	[60]

### 3.5. Quality Assessment and Risk of Bias

**Figure 7:**
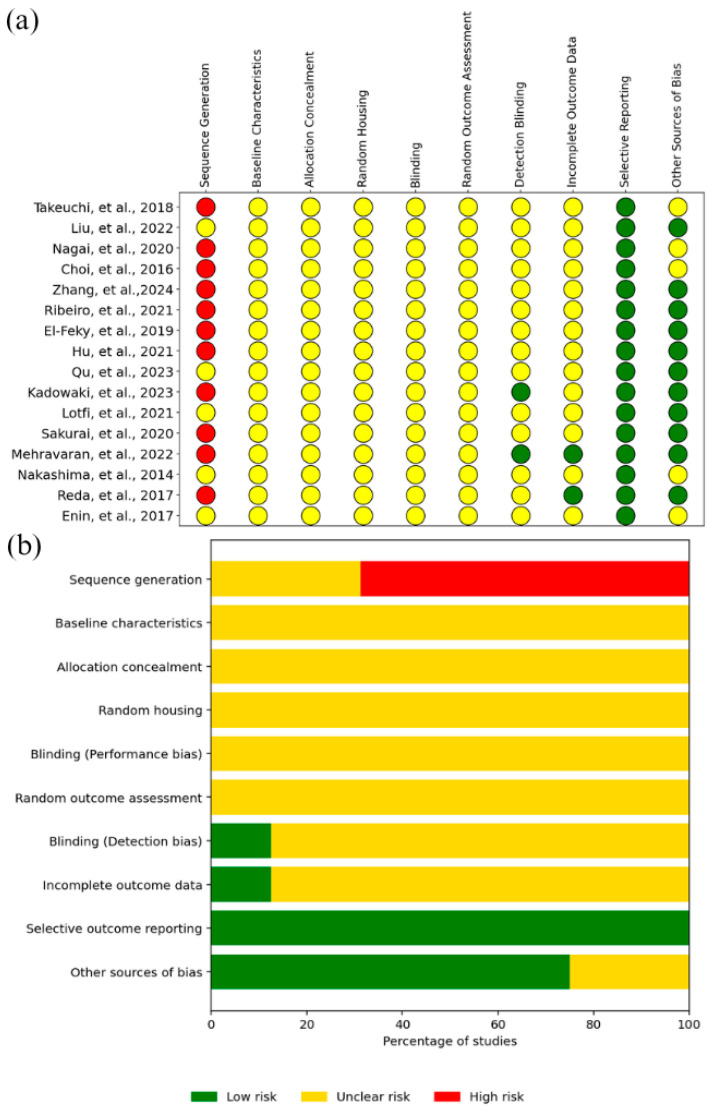
Risk of bias analysis of in vivo studies (SYRCLE) [44,45,46,47,48,49,50,51,52,53,54,55,56,57,58,59]. (**a**) SYRCLE analysis on individual studies. Red dots indicate high risk, yellow dots indicate uncertain risk, and green dots indicate low risk in each domain; (**b**) Summary graph of SYRCLE analysis.

The risk of bias assessment for in vivo studies was conducted using the SYRCLE criteria and is summarized in Figure 7. Figure 7a shows a traffic-light plot, in which each row corresponds to an in vivo study included in this review, and each column corresponds to one of the ten bias domains. Red dots indicate high risk, yellow dots indicate uncertain risk, and green dots indicate low risk in each domain. In Figure 7b, a column chart shows, on the x-axis, the percentage of studies in each risk category, and on the y-axis, the respective domains assessed.

Overall, 73% of the evaluations were at an uncertain risk, 20% were at low risk, and 7% were at high risk. Sequence generation had the highest incidence of high risk, while selective outcome reporting had the most low-risk assessments.

**Figure 8:**
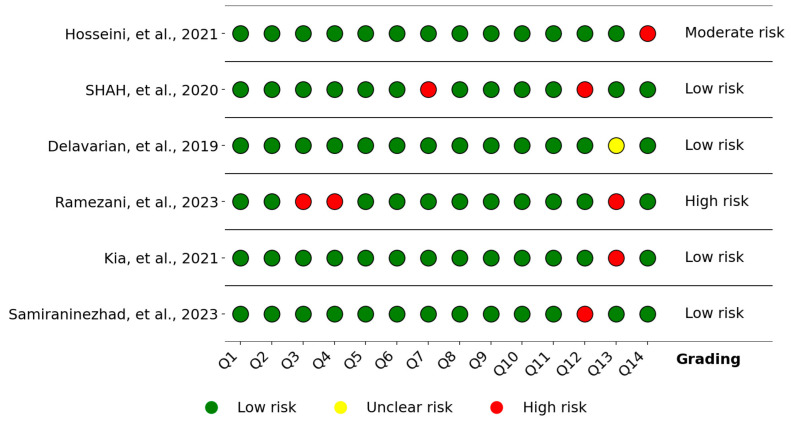
Risk of bias graph of clinical trial studies (NIH) [19,30,32,33,34,60].

For clinical studies, the risk of bias was assessed using the NIH’s Quality Assessment of Controlled Intervention Studies tool, as described in the Methodology section, and the results are summarized in Figure 8. In Figure 6, Q1 to Q14 identify the domains analyzed, and the last column presents the overall risk, adapted according to [61]: two high-risk domains were classified as moderate risk, and three or more as high risk. According to this criterion, four studies were classified as low risk, while only one study was considered high risk due to lack of allocation concealment, lack of blinding of participants and providers, and lack of pre-specification of subgroups, although this same study presented low risk in the other domains.

## 4. Discussion

DDSs are designed to administer therapeutic agents in a controlled and targeted way. Emulsions, suspensions, micelles, liposomes, hydrogels, nanofibers, dendrimers, NPs, and stimuli-responsive polymers protect drugs from degradation, increase their bioavailability, and reduce side effects. Studies of these platforms typically proceed through in vitro evaluations, in vivo experiments, and clinical trials.

### 4.1. Drug Delivery Systems

In this review, from the analysis of the 32 articles on micro and nano DDS, 26 articles utilized polymeric-based DDS, highlighting polymer versatility and properties such as easy modification, biocompatibility, mucoadhesion, viscosity, and swelling capacity. Only one study [39] presented lipid-based DDS utilizing solid lipid NPs, known for their safety. Two studies employed nanocrystals [46,57]; those DDSs are known for their low-price development, low toxicity, and simplicity. Additionally, two studies [54,59] explored nanoemulsions, and a single study referenced a patented DDS [32]. Among the in vivo and clinical trials presented in the articles, the in situ administration route was the predominant method, employed in most studies. Three studies [30,33,34] utilized oral administration, while another adopted the intraperitoneal route [54]. A single investigation [45] compared both intravenous and in situ delivery of cannabidiol nanomicelles and free cannabidiol to evaluate their differences, and the authors found similar wound closure and tissue regeneration at both administration routes after 4 days of treatment. The preference for in situ administration reveals its relevance in targeted drug delivery for oral mucositis, due to its localized therapeutic effects, avoidance of first-pass metabolism, reduction on toxic effects, and higher patient compliance [62].

An important characteristic to enhance DDS therapeutic activity on OM is nanoparticle size, which is known to enhance important characteristics such as mucoadhesiveness, cellular uptake, water solubility and controlled drug release [63]. In the study [46], the authors developed hydrogels incorporating nanocrystals resulting in enhanced adhesiveness and faster drug release rate. Those results led to a 25-fold higher rebamipide drug concentration in the lesion area and a 2.7-fold wound size reduction when compared to microcrystals. Similar results on the effect of nanoparticle size were found in [53,57] where there was enhanced OM treatment with NPs containing the same compounds, and NPs with reduced size promoted faster and more efficient wound healing. In an in vitro study, Kawano et al. [40] also observed an improvement in artificial mucosal retention, water solubility, and nanoparticle stability with smaller sized NPs. These results could suggest that nanosystems are more efficient in the treatment of OM than microsystems.

Nanoparticle modification, such as coating with mucoadhesive polymers, can alter the physicochemical properties affecting therapeutic efficacy on mucous tissue by enhancing bioavailability, rate of drug release, and time of retention on the tissue. Several studies investigated this strategy to increase the efficiency of DDSs in the treatment of OM.

Chitosan was the most utilized polymer in this strategy. In studies [35,44,47] the authors explored how the modification of a DDS with chitosan modifies the DDS’ interaction with the glycoprotein mucin present on the oral mucus layer. This factor is mostly explained by the positive charges presented on the chitosan molecule at an acid and neutral pH; this resulted in a 2.3-fold increase in retention time of NPs in oral mucosa and an in vivo retention on mice oral mucosa of up to 6 h only for chitosan-coated NPs. Those results led to a decrease in time, 3.6 days and 5.2 days, to reduce the ulceration area of OM in a mice model when compared to naked PLGA NPs and rebamipide suspension [44].

Other polymers such as PDA and PVA were also explored by authors to enhance NPs’ therapeutic effect on OM. PDA is a hydrophilic and negatively charged molecule capable of coating NPs and enhancing their mucus-penetrating properties; this promotes an increase in wound closure from 62.95 ±  9.83% to 91.51  ±  9.63% derived from non-PDA and PDA-containing DDSs tested on OM model rats [51]. Similarly, an increase in wound closure from 65.27% to 80.60% was also found when comparing, respectively, drug delivery systems without or with mesoporous PDA [52].

In the work of Takeuchi and colleagues [44], PLGA NPs coated with chitosan were used to deliver rebamipide, resulting in enhanced mucoadhesive properties and sustained release, which significantly reduced the ulcerated area and accelerated recovery in mice. Another study [49] demonstrated that PLGA NPs encapsulating dexamethasone effectively reduced the levels of inflammatory markers (IL-1β and TNF-α) and preserved tissue integrity in experimental models. On the other hand, *P. indica* extract NPs stabilized with Pluronic F127 was developed as an oral spray, which promoted cell migration and wound healing, although with lower efficacy than the pure extract at high concentrations (125 µg/mL) [31].

Innovative approaches have been proposed to enhance mucoadhesive adhesion and bioavailability. Hu et al. [51] produced a mussel-inspired mucoadhesive film containing PLGA NPs coated with PDA that increase the bioavailability of dexamethasone by 3.5 times and accelerated wound closure in rats, outperforming commercial formulations. Similarly, Qu and coworkers [52] developed microneedles containing MPDA NPs loaded with TA, ensuring controlled release, reducing inflammation, and promoting epithelial regeneration in OM models. In work of Kadowaki and collaborators [53], the authors formulated mucoadhesive hydrogels with troxipide NPs, which, through CME, accelerated wound healing in hamsters, demonstrating superiority over conventional microparticles.

Micelles have emerged as promising alternatives in the treatment of OM. Fucoidan micelles loaded with cannabidiol (CBD) prepared to target the drug to inflamed sites, resulting in a reduction in the ulcerated area and inflammatory cell infiltration in mice [45]. The formulation provided controlled release and prolonged retention of CBD, accelerating healing. Delavarian at al. [32] utilized curcumin micelles in patients undergoing radiotherapy, resulting in a delay in the onset of symptoms and a reduction in the severity of OM, demonstrating high bioavailability and strong anti-inflammatory action.

Other nanostructured systems include nanofibers and nanogels, both with mucoadhesive properties and controlled release. Choi and colleagues [47] reported that nanofibers composed of Eudragit L100 and chitosan, which contain hGH, promoted the complete regeneration of ulcers in dogs, ensuring sustained drug release. Tort and Acartürk [36] developed nanofibers of sodium alginate and PEO loaded with glutamine, which demonstrated good adhesion to the oral mucosa and gradual drug release. In another approach, starch and PEGDE nanogels containing vancomycin exhibited rapid release of the antibiotic and strong activity against pathogens related to OM, making them promising options for the control of oral infections [37].

Hydrogels and mucoadhesive films have been explored for prolonged drug release and protection of the oral mucosa. Campos and colleagues [43] developed in situ-forming hydrogels from buccal films and tablets, which exhibited high mucoadhesive capacity and sustained release of budesonide and lidocaine. Enin and coworkers [59] created bilayer buccal films containing nanoemulsions of diclofenac and lidocaine, providing anti-inflammatory action and effective analgesia, as well as good patient acceptance. In another study, hydrogels containing rebamipide nanocrystals demonstrated high tissue retention and strong local therapeutic effects in hamster mucositis models, promoting accelerated epithelial regeneration and reducing inflammation via CME [46].

### 4.2. In Vitro Studies

In vitro assays represent the initial step in evaluating the potential of micro- and nanosystems for the treatment of a given disease. This stage involves the assessment of cytotoxicity, release kinetics, stability under physiological conditions, mucoadhesion, and diffusion in oral epithelial models. These tests establish the biocompatibility of the systems, provide insight into mechanisms such as cellular uptake and barrier penetration, and guide the rational selection of candidates for subsequent in vivo evaluation.

Examples of nanofiber-based systems evaluated at this stage include those described by Tort and Acartürk [36] and Halder et al. [38]. In work of Tort and Acartürk [36], glutamine-loaded nanofibers released over 85% of the drug within 4 h and remained stable at 4 °C and 25 °C; physical changes were observed only at 40 °C and 75% relative humidity, suggesting their applicability in oral mucositis therapy. Halder et al. [38] prepared artificial saliva composed of hyaluronic acid nanofibers loaded with glycyrrhizin that had a moisturizing effect of 86.16%, antimicrobial and anti-inflammatory properties, biocompatibility, and six-month stability. Glycyrrhizin has a chemoprotective effect, whereas hyaluronic acid promotes cell regeneration, contributing to a reduction in inflammation, xerostomia, and infections.

One topical approach involves an oral spray formulated with NPs of *P. indica* extract; the preparation promoted the cell migration needed for wound closure, maintained colloidal stability, and passed accelerated and microbiological stability tests, confirming its efficacy and safety for oral use [31]. Another strategy applies cross-linked starch nanogels with PEGDE as carriers for vancomycin in bacterial infections associated with oral mucositis: the system reached 88.1% encapsulation efficiency; released 70% of the antibiotic in 15 min and the full dose in 60 min; biodegraded within 2 h; and inhibited S. aureus, S. pyogenes, and S. mutans more effectively than free vancomycin [37]. A further example is a mucoadhesive gel containing solid lipid NPs loaded with N-acetylglucosamine; the formulation adhered well to the mucosa, was biocompatible, stimulated cell proliferation, preserved epithelial integrity, and performed on par with Carbopol 934 at 2%, indicating safety and clinical potential for managing oral lesions [39].

Studies using microparticulate systems have also demonstrated their efficacy in vitro for the treatment of oral mucositis. The encapsulation of plant extracts, as shown in the works with Actinidia arguta [42] and chestnut shell [41], indicated that natural compounds protected by suitable matrices maintain stability, allow for controlled release, and preserve biocompatibility. The use of chitosan in one study [35] highlighted its mucoadhesive properties and its capacity to modulate drug release, thereby extending mucosal contact and improving therapeutic efficacy. Together, these findings emphasize the potential of microparticles and microspheres as platforms for formulations designed to treat oral lesions.

Hydrogels and mucoadhesive films have been explored for prolonged drug release and protection of the oral mucosa. In situ forming hydrogels developed from buccal films and tablets exhibited high mucoadhesive capacity and sustained release of budesonide and lidocaine [43].

Finally, the rebamipide nanoparticle mouthwash prepared by wet milling with HPC and SLS exhibited enhanced drug solubility and stability, whereas the HPC–SLS polymers provided mucoadhesive properties that prolong the residence time on the oral mucosa [40].

### 4.3. In Vivo Studies

In rat models, Hu et al. [51] used Sprague Dawley rats and a PVA-PDA mucoadhesive film and reported strong mucoadhesive adhesion, enhanced mucosal penetration, increased drug bioavailability, significant improvement in wound closure, complete epithelial regeneration, and high biocompatibility. Qu and colleagues [52] similarly utilized Sprague Dawley rats and microneedle patches loaded with TA@MPDA-HA/BSP and reported a significant reduction in inflammation (decrease in TNF-α), accelerated epithelial regeneration, and rapid recovery from oral ulcers. Nakashima team [57] used rats in models of cauterization-induced oral ulcers and radiation-induced glossitis, and submicronized rebamipide formulations promoted a significant reduction in the ulcerated area, suggesting epithelial regeneration and acceleration of recovery time.

In hamster models, OM is primarily induced by acetic acid [46,53] or by the combination of 5-FU and mechanical trauma [49]. Treatment using rebamipide nanocrystal gel in hamsters with OM induced with acetic acid promoted a significant reduction in inflammation, accelerated epithelial regeneration, and resulted in a shorter recovery time, with a smaller wound area on the third day and reduced migration of inflammatory cells [46]. Consistent with these findings, Kadowaki and collaborators [53] also used hamsters with acetic acid-induced OM and reported that a troxipide nanoparticle hydrogel significantly improved wound healing, indicating superior epithelial regeneration, reduced inflammation, and faster recovery time. In a different hamster model, OM was induced with 5-FU and mechanical trauma, and treatment with dexamethasone-loaded PLGA NPs also led to a significant reduction in inflammation, accelerated epithelial regeneration, less inflammatory infiltration, and a shorter recovery time [49].

Studies in mice explored various formulations and OM induction models. Takeuchi team [44] used mice with chemotherapy-induced OM and demonstrated that PLGA NPs loaded with rebamipide and coated with chitosan resulted in a significant reduction in the ulcer area and a 3.6-day recovery time. Liu and coworkers [45] induced OM in mice with 5-FU and acetic acid, and treatment with CBD micelles promoted a significant reduction in inflammation, improved oral ulcer healing with a reduced ulcer area, and promoted faster recovery. Lofti at al. [54] also used male albino mice and reported that quercetin nanoemulsions led to a significant reduction in oral lesions, decreased MDA, increased SOD and CAT, decreased inflammatory infiltration and tissue degradation, promoted epithelial regeneration, and reduced recovery time. Zhang et al. [48] used Kunming mice with chemotherapy-induced OM and demonstrated that Apr@PMPB@S buccal tablets promoted a significant reduction in inflammation (a decrease in proinflammatory cytokines), remarkable epithelial regeneration, and a reduced recovery time. In the work of Sakurai et al. [55], although not directly focusing on inflammation and regeneration, the authors used mice to evaluate an indomethacin microparticle gel and suggested that it may alleviate OM pain, which implies a therapeutic effect on the inflammatory condition.

In albino rabbits, OM induced with acetic acid and treated with a thermosensitive and mucoadhesive BZN-PLGA-NP-HG resulted in reduced inflammation (a decrease in the ulcer area), accelerated epithelial regeneration, and a reduced recovery time of 10 days [50].

Reda and collaborators [58] also used rabbits with induced OM, and nanofibers loaded with ketoprofen demonstrated a significant reduction in inflammation (decrease in inflammatory infiltration and epithelial re-epithelialization) and accelerated recovery time, with notable efficacy in reducing the clinical severity of mucositis.

In beagle dogs, Choi and colleagues [47] evaluated chitosan-coated nanofiber sheets with hGH for healing ulcers in the oral mucosa. The results revealed a reduction in inflammation, increased cell proliferation, and accelerated epithelial regeneration. Notably, the sheets coated with 0.5% chitosan provided complete healing of ulcers in 7 days, which was a significantly faster recovery time than that of the control groups.

### 4.4. Clinical Trial Studies

The few available clinical studies corroborate the potential of nanoformulations to delay the onset and mitigate the severity of OM but reveal limitations that prevent their immediate clinical adoption. To date, no microstructured system has advanced to patient trials in the search criteria used in this review.

The use of curcumin in mouthwash, micelles, and nanocapsules has been extensively investigated. Overall, these studies indicate that curcumin reduces inflammation and the severity of mucositis, although its limited bioavailability still poses a challenge. In the study by Shah and coworkers [30], curcumin mouthwash with NPs demonstrated the advantage of delaying the onset of radiation-induced OM compared with benzydamine, reinforcing the potential of this delivery system. However, the need for larger studies, the high rate of follow-up loss, and the complexity in formulation hinder immediate clinical replication. Similarly, studies with curcumin micelles and nanocapsules confirmed their efficacy but highlighted challenges such as formulation standardization and the need for larger clinical trials [32,33,34].

Another investigated system was the chitosan-based doxepin nanoformulation, which demonstrated greater efficacy in reducing the severity of mucositis than conventional treatment with diphenhydramine and aluminum–magnesium hydroxide mouthwash. The combination of chitosan and doxepin potentiated the anti-inflammatory and analgesic effects, leading to faster patient recovery. However, its clinical implementation depends on evaluating its efficacy in different populations and the feasibility of large-scale production [60].

Finally, the silymarin solution presented theoretical benefits, such as increased bioavailability, but did not demonstrate significant clinical efficacy in the current study. Factors such as formulation instability, adverse reactions possibly associated with concomitant chemotherapy, and the absence of direct comparisons with conventional drugs may have contributed to these results [19].

These findings support the potential of nanostructured systems for the treatment of OM, with advantages which include controlled release, improved mucosal retention, and prolonged action. However, challenges such as formulation stability, dose standardization, clinical reproducibility, and large-scale production still need to be overcome before these technologies can be widely used in clinical practice.

### 4.5. Limitations of the Review

The objective of this review was to compile articles related to the use of organic, non-metallic micro- and nanosystems in the treatment of OM. The application of inorganic NPs for OM was partially discussed in article [27]. Most of the analyzed studies are in the preclinical (in vitro or in vivo) phase, with a limited number progressing to clinical trials. This indicates a promising potential for these technologies but also highlights the need for more robust validations to increase their clinical viability. Another limitation is that several studies showed a risk of bias, indicating the need for further research with lower bias levels. In addition, the small number of studies and diversity of systems restricts the breadth of the discussion. Nevertheless, the gathered content provides a consistent overview of the published advances in the field.

## 5. Conclusions

In the articles analyzed for this review, researchers used both synthetic and natural polymers. Natural polymers, including chitosan, hyaluronic acid, and sodium alginate, stand out for their intrinsic properties, including biocompatibility, biodegradability, and high mucoadhesiveness. These polymers are generally used where prolonged retention and controlled release in mucosal tissues are needed. Despite their advantages, they face challenges related to batch-to-batch variability and purification processes that can limit their predictability in large-scale clinical applications.

On the other hand, synthetic polymers such as PLGA, Eudragit, and HPMC offer greater control over mechanical properties, degradation rates, and predictability of results. These characteristics make these materials a preferred choice for formulations that require stability and sustained drug release. Their versatility and adaptability make them ideal for developing more robust and customizable systems. Furthermore, the integration of natural and synthetic polymers in hybrid formulations has emerged as a promising trend, identified in eight studies in this review, combining the advantages of both types of polymers to maximize therapeutic efficacy and reduce adverse effects.

In terms of the stage of research development, most of the studies analyzed are in the preclinical phase, with a limited number of studies advancing to clinical trials. This scenario illustrates the promising potential of technologies based on micro- and nanosystems, especially in terms of therapeutic efficacy and in reducing adverse effects and highlights the need for future studies to address these limitations to facilitate the translation of these promising technologies into clinical practice.

Although this study did not fully explore the potential of micro- and nanodrug delivery systems for the treatment of OM, owing to its integrative review nature, there is growing scientific interest in the application of these systems to optimize this treatment. Importantly, owing to advances in nanotechnology, publications in this area are still limited, reflecting its emerging state in scientific and technological research.

## Data Availability

Data are available upon request.
